# Clinical relevance of breast cancer-related genes as potential biomarkers for oral squamous cell carcinoma

**DOI:** 10.1186/1471-2407-14-324

**Published:** 2014-05-07

**Authors:** Toshima Z Parris, Luaay Aziz, Anikó Kovács, Shahin Hajizadeh, Szilárd Nemes, May Semaan, Chang Yan Chen, Per Karlsson, Khalil Helou

**Affiliations:** 1 Department of Oncology, Institute of Clinical Sciences, Sahlgrenska Cancer Center, Sahlgrenska Academy at University of Gothenburg, Gothenburg, Sweden; 2 Department of Otolaryngology, Institute of Clinical Sciences, Sahlgrenska Academy at University of Gothenburg, Gothenburg, Sweden; 3 Department of Clinical Pathology and Cytology, Sahlgrenska University Hospital, Gothenburg, Sweden; 4 Division of Clinical Cancer Epidemiology, Department of Oncology, Institute of Clinical Sciences, Sahlgrenska Academy at Gothenburg University, Gothenburg, Sweden; 5 Regional Cancer Center (West), Western Sweden Health Care Region, Sahlgrenska University Hospital, Gothenburg, Sweden; 6 Department of Radiation Oncology, Beth Israel Deaconess Medical Center, Harvard Medical School, Boston, Massachusetts, USA

**Keywords:** Oral squamous cell carcinoma, Outcome prediction, Molecular biomarker, Immunohistochemistry, Model validation

## Abstract

**Background:**

Squamous cell carcinoma of the oral cavity (OSCC) is a common cancer form with relatively low 5-year survival rates, due partially to late detection and lack of complementary molecular markers as targets for treatment. Molecular profiling of head and neck cancer has revealed biological similarities with basal-like breast and lung carcinoma. Recently, we showed that 16 genes were consistently altered in invasive breast tumors displaying varying degrees of aggressiveness.

**Methods:**

To extend our findings from breast cancer to another cancer type with similar characteristics, we performed an integrative analysis of transcriptomic and proteomic data to evaluate the prognostic significance of the 16 putative breast cancer-related biomarkers in OSCC using independent microarray datasets and immunohistochemistry. Predictive models for disease-specific (DSS) and/or overall survival (OS) were calculated for each marker using Cox proportional hazards models.

**Results:**

We found that CBX2, SCUBE2, and STK32B protein expression were associated with important clinicopathological features for OSCC (peritumoral inflammatory infiltration, metastatic spread to the cervical lymph nodes, and tumor size). Consequently, SCUBE2 and STK32B are involved in the hedgehog signaling pathway which plays a pivotal role in metastasis and angiogenesis in cancer. In addition, CNTNAP2 and S100A8 protein expression were correlated with DSS and OS, respectively.

**Conclusions:**

Taken together, these candidates and the hedgehog signaling pathway may be putative targets for drug development and clinical management of OSCC patients.

## Background

Oral squamous cell carcinoma (OSCC) is the most common malignancy form in the head and neck region, accounting for about 260,000 new cases and 124,000 OSCC-related deaths worldwide annually [1,2]. In western countries, the etiology of some newly diagnosed primary SCCs of the head and neck has shifted from tobacco and alcohol abuse to human papillomavirus (HPV) infections, possibly as a result of changes in sexual practices [3-8]. Despite aggressive treatment modalities, 5-year survival rates for advanced head and neck cancers have remained low and relatively unchanged (about 50-60%) for several decades, partially due to early locoregional recurrences within 2 years of initial treatment [9]. There is therefore a pressing need for molecular predictors that enable earlier detection of the disease, describe tumor behavior, and improve risk assessment to identify patients at risk for recurrence and OSCC-related death.

Molecular profiling has become a common and effective method for cancer gene discovery and classification of cancer. Almost a decade ago, Chung *et al*. identified four intrinsic subtypes for head and neck squamous cell carcinoma with clinical and biological implications [10]. Consequently, one of the subtypes with the most unfavorable prognosis also displayed strikingly similar transcriptional patterns with the breast carcinoma basal-like phenotype and lung squamous cell carcinoma. Recently, additional evidence of shared cellular processes was found between breast carcinoma and oral squamous cell carcinoma, i.e. mechanisms for tumor lymphangiogenesis and metastasis to the regional lymph nodes as well as HER2/*neu* polymorphisms [11,12]. These findings suggest that cancers derived from different sites of origin may perturb common signaling pathways and thereby display similar tumor characteristics [13]. To test this hypothesis, we evaluated the prognostic potential of 16 putative prognostic biomarkers (*AZGP1*, *BTG2*, *CBX2*, *CNTNAP2*, *DNALI1*, *LOC389033*, *NME5*, *PIP*, *S100A8*, *SCUBE2*, *SERPINA11*, *STC2*, *STK32B*, *SUSD3*, *UBE2C*, and *WHSC1L1*) previously identified in breast carcinoma using oral squamous cell carcinoma [14,15]. Interestingly, several of the putative biomarkers have been implicated in the carcinogenesis of more than one cancer form. We, and others, have also been able to show that *AZGP1*, *S100A8*, and *STK32B*, as well as *CNTNAP2* are associated with the basal-like phenotype and lymph node metastasis, respectively [15-17].

Here, we investigated the prognostic potential of the gene expression signature in relation to clinical outcome, disease-specific survival (DSS) and/or overall survival (OS), in two steps. First, transcriptional levels for each gene were evaluated with respect to the clinical endpoints using publicly available Affymetrix one-channel microarray (n = 168) and Illumina RNASeq datasets (n = 198) for OSCC from the Gene Expression Omnibus (GEO) and The Cancer Genome Atlas (TCGA) repositories, respectively. Second, because correlation between mRNA/protein levels is frequently low, Cox proportional hazards models for DSS and OS were also calculated using immunohistochemical protein expression patterns from 43 OSCC cases together with established clinicopathological features (tumor size and node status or tumor size and age, respectively).

## Methods

### Patient cohorts

To evaluate the prognostic potential of the *AZGP1*, *BTG2*, *CBX2*, *CNTNAP2*, *DNALI1*, *LOC389033*, *NME5*, *PIP*, *S100A8*, *SCUBE2*, *SERPINA11*, *STC2*, *SUSD3*, *STK32B*, *UBE2C*, and *WHSC1L1* genes in OSCC specimens, three patient cohorts were compiled primarily from squamous cell carcinomas of the oral cavity. External gene expression datasets and corresponding clinical information for Cohorts I-II were compiled from the Gene Expression Omnibus (GEO) and The Cancer Genome Atlas (TCGA) repositories, respectively. Cohort I included two Affymetrix U133 Plus 2.0 GeneChip datasets (GEO accession numbers GSE41613 and GSE42743) containing 168 OSCC samples (oropharynx samples were excluded from the analysis) [18]. Cohort II consisted of normalized RNAseq by Expectation-Maximization (RSEM) gene datasets from 198 OSCC patients (oral cavity: buccal mucosa, floor of mouth, tongue), which were downloaded from The Broad Institute TCGA GDAC (http://gdac.broadinstitute.org/runs/stddata__2014_01_15/). Cohort III consisted of 43 OSCC cases originating from the oral cavity (buccal gingiva, floor of mouth, tongue), which had been diagnosed between 1997-2004 at Sahlgrenska University Hospital in Gothenburg, Sweden. All patients underwent diagnostic battery inclusive biopsy of the primary tumor, palpation of the neck, radiological examination with MRT and/or CT, and TNM classified according to the American Joint Committee on Cancer (AJCC) staging system. Surgical excision of the primary tumor and supraomohyoid neck dissection (SOHND) were performed. In total, 16 patients had cervical lymph node metastases (pN1) of which 5/16 patients had micrometastases (pNmic) as assessed using anti-human monoclonal cytokeratin AE1/AE3, and 27 patients were lymph node-negative (pN0). Lymph node-positive patients received post-operative radiotherapy to the neck, whereas pN0 and pNmic patients were followed up clinically. All patients were followed up for at least five years during which seven patients (16%) developed local and/or regional recurrence, including two patients with pN1, three patients with pNmic, and two patients with pN0 disease. Three of the five patients with micrometastases developed recurrence (60%), of which two (40%) died within three years due to OSCC-related causes. The clinicopathological features for Cohorts I-III are summarized in Table 1.

**Table 1:** **Clinicopathological features for OSCC patients in Cohorts I**-**III**

	**Cohort I† (n = 168)**	**Cohort II‡ (n = 198)**	Cohort III* (n = 43)
*Age* (*y*)			
19-39	13 (8%)	11 (6%)	2 (5%)
40-49	29 (17%)	24 (12%)	4 (9%)
50-59	43 (26%)	50 (25%)	12 (28%)
60-88	83 (49%)	113 (57%)	25 (58%)
Not available	0 (0%)	0 (0%)	0 (0%)
*Sex*			
Female	47 (28%)	68 (34%)	20 (47%)
Male	121 (72%)	130 (66%)	23 (53%)
Not available	0 (0%)	0 (0%)	0 (0%)
*Tumor site*			
Oral cavity	71 (42%)	198 (100%)	43 (100%)
Not available	97 (58%)	0 (0%)	0 (0%)
*Cervical lymph node status*			
pN0	0 (0%)	70 (35%)	27 (63%)
pN1	0 (0%)	98 (50%)	16 (37%)
Not available	168 (100%)	30 (15%)	0 (0%)
*Tumor size*			
T1-T2	30 (18%)	83 (42%)	36 (84%)
T3-T4	41 (24%)	111 (56%)	7 (16%)
Not available	97 (58%)	4 (2%)	0 (0%)
*Clinical stage*			
I/II	71 (42%)	61 (31%)	25 (58%)
III/IV	97 (58%)	133 (67%)	18 (42%)
Not available	0 (0%)	4 (2%)	0 (0%)
*Differentiation*			
Well	0 (0%)	30 (15%)	12 (30%)
Moderate	0 (0%)	128 (65%)	22 (51%)
Poor	0 (0%)	38 (19%)	7 (16%)
Not available	168 (100%)	2 (1%)	0 (0%)
*Tumor inflammatory infiltration*			
Minimal	0 (0%)	0 (0%)	13 (30%)
Moderate	0 (0%)	0 (0%)	13 (30%)
Strong	0 (0%)	0 (0%)	16 (37%)
Not available	168 (100%)	198 (100%)	0 (0%)
*Tobacco smoking history*			
Never smoker	15 (9%)	46 (23%)	18 (42%)
Former smoker	28 (17%)	87 (44%)	5 (12%)
Current smoker	28 (17%)	57 (29%)	12 (28%)
Chewing tobacco	0 (0%)	0 (0%)	1 (2%)
Not available	97 (58%)	8 (4%)	7 (16%)
*p16*			
Negative	97 (58%)	0 (0%)	35 (81%)
Positive	0 (0%)	0 (0%)	8 (19%)
Not available	71 (42%)	198 (100%)	0 (0%)

†Gene Expression Omnibus (GEO) accession numbers GSE41613 and GSE42743.

‡HNSC The Cancer Genome Atlas (TCGA) data.

*FFPE samples from Sahlgrenska University Hospital, Gothenburg, Sweden.

### Immunohistochemistry

For Cohort III, 45 FFPE samples corresponding to the 43 patients were obtained from the Department of Pathology at Sahlgrenska University Hospital and used in immunohistochemistry experiments in accordance with the Declaration of Helsinki and approved by the Medical Faculty Research Ethics Committee (Gothenburg, Sweden). The ethics committee approved a waiver of written consent to use the tumor specimens in the study. Histological classification and TNM staging of the tumor specimens were performed according to the WHO classification and International Union Against Cancer (UICC), respectively [19,20]. Optimal antibody dilutions and assay conditions were achieved for immunohistochemistry using OSCC as positive controls. Four micrometer full-face FFPE sections were pretreated using the Dako PTLink system (Dako, Carpinteria, CA, USA) and processed using the Dako Envision™ FLEX High pH Link Kit (pH 9) for p16, AZGP1, BTG2, CBX2, CNTNAP2, NME5, S100A8, SCUBE2, SERPINA11, STC2, SUSD3, STK32B, SUSD3, UBE2C, and WHSC1L1 as listed in Additional file 1: Table S1. Peroxidase-catalyzed diaminobenzidine was used as the chromogen, followed by hematoxylin counterstain. The slides were then rinsed with deionized water, dehydrated in absolute alcohol, followed by 95% alcohol, cleared in xylene, and mounted. H & E staining was performed on one FFPE section to facilitate histological assessment. The degree of lymphoplasmacytic infiltration (inflammatory infiltration) was classified as minimal (few inflammatory cells), moderate (1-2 mm margin), and strong (>2 mm margin) according to the density of inflammatory cells. Immunostaining was evaluated by a head and neck pathologist, blinded to patient clinical outcome, and scored as previously described using the semi-quantitative H-score method to calculate the sum of the percentage and intensity of positively stained tumor cells within the invasive tissue component (negative staining = 0; weak staining = 1+; moderate staining = 2+; strong staining = 3+). The H-score ranged from 0 to 300, where H-score = (1 ×%1+) + (2 ×%2+) + (3 ×%3+) [21]. The X-tile software (version 3.6.1) was used to determine an H-score cut-off for positive staining by dichotomizing patients according to H-score value and clinical outcome, as listed in Additional file 1: Table S1 [22]. FFPE specimens lacking an invasive tissue component were removed from the analysis. Each tumor specimen was scored once, where multiple FFPE sections representing the same tumor were averaged. Staining was evaluated in the invasive and peritumoral stromal/normal tissue components.

### Statistical analysis

Statistical analyses were performed using a 0.05 *P*-value cutoff (two-sided) in R/Bioconductor (version 2.15.0). Putative prognostic biomarkers for OSCC were identified in two steps. First, the prognostic potential of aberrant biomarker gene expression was evaluated in external microarray and RNASeq datasets (Cohorts I-II). Then, predictive models for DSS and OS were developed using biomarker protein expression (Cohort III).

####  Evaluation of gene expression patterns for the 16-marker signature in external microarray and RNASeq datasets

Univariate Cox proportional hazard models were calculated for each gene using the endpoints disease specific-survival (DSS) and/or overall survival (OS). OSCC survival rates were defined as a) the period from initial diagnosis to OSCC-related death for DSS and b) period from initial diagnosis to death from any cause for OS. Data processing and Cox regression analysis of the Affymetrix one-channel microarray datasets (Cohort I) and normalized RNASeq RSEM values (Cohort II) were performed using Nexus Expression 3.0 (BioDiscovery).

#### Development of a predictive model for DSS and OS using protein expression

Survival rates (DSS and OS) at different protein expression levels were depicted with Kaplan-Meier curves and tested with log-rank test. The relationship between clinicopathological features and protein expression was evaluated using two-tailed Fisher’s exact test. Multivariate analysis was conducted using the Cox proportional hazard model for DSS or OS with stepwise selection to assess the predictive strength and additive accuracy of protein expression after adjusting for established clinicopathological features (tumor size and node status or tumor size and age, respectively). A concordance index (C-index) for the time-dependent area under the ROC curve (AUC (t)) was calculated to assess model predictive performance, varying from C-index = 0.5 (no predictive power) to C-index = 1 (perfect prediction).

## Results

### Prognostic potential of the molecular biomarkers in external gene expression microarray and RNASeq datasets

In previous work, we showed the clinical significance of 16 candidate molecular biomarkers (*AZGP1*, *BTG2*, *CBX2*, *CNTNAP2*, *DNALI1*, *LOC389033*, *NME5*, *PIP*, *S100A8*, *SCUBE2*, *SERPINA11*, *STC2*, *STK32B*, *SUSD3*, *UBE2C*, and *WHSC1L1*) in invasive breast carcinoma [14,15,23]. To investigate whether these putative prognostic biomarkers may also play a pivotal role in the aggressive nature of OSCCs, the effect of altered gene expression patterns on clinical outcome was evaluated using two external OSCC patient cohorts (Cohorts I-II; Table 1). Cox proportional hazard models were calculated for each gene with relation to clinical endpoints (DSS and/or OS; Table 2).

**Table 2:** **Univariate Cox proportional hazard regression models for OSCC Cohorts I**-**II**

		**Cohort I (n = 168)**				**Cohort II (n = 198)**	
		**Disease-specific survival**		**Overall survival**		**Overall survival**	
**No.**	**Variables**	**Coefficient**	**p-value**	**Coefficient**	**p-value**	**Coefficient**	**p-value**
*1*	*AZGP1*	-0.210	**0.001**	-0.058	0.151	-0.020	0.632
*2*	*BTG2*	-0.651	**0.020**	-0.597	**0.005**	-0.036	0.807
*3*	*CBX2*	0.334	0.313	0.690	**0.005**	0.061	0.591
*4*	*CNTNAP2*	0.225	0.389	0.262	0.176	-0.003	0.938
*5*	*DNALI1*	-0.348	0.617	-0.246	0.638	-0.077	0.429
*6*	*LOC389033*	ND	ND	ND	ND	-0.464	0.142
*7*	*NME5*	-0.302	0.477	0.299	0.323	0.225	0.050
*8*	*PIP*	-0.242	**0.010**	-0.003	0.948	-0.009	0.871
*9*	*S100A8*	-0.125	0.125	-0.121	0.053	-0.103	0.072
*10*	*SCUBE2*	-0.625	**0.026**	-0.250	0.195	-0.130	0.067
*11*	*SERPINA11*	ND	ND	ND	ND	-0.036	0.625
*12*	*STC2*	0.561	**0.001**	0.459	**0.001**	0.082	0.379
*13*	*STK32B*	0.488	0.303	0.850	**0.015**	-0.038	0.612
*14*	*SUSD3*	1.448	0.080	1.092	0.088	0.050	0.613
*15*	*UBE2C*	0.279	**0.042**	0.166	0.107	0.388	**0.005**
*16*	*WHSC1L1*	-0.266	0.368	0.014	0.942	0.010	0.949

Statistically significant variables (p < 0.05) are displayed in bold text.

*Abbreviation*: *ND* Not determined.

In Cohort I, two genes (*LOC389033* and *SERPINA11*) were not found on the Affymetrix platform and therefore excluded from the analysis. Univariate Cox regression analysis showed that low levels of *AZGP1* (*P* = 0.001), *BTG2* (*P* = 0.020), *PIP* (*P* = 0.010), and *SCUBE2* (*P* = 0.026) were indicative of a more unfavorable prognosis, whereas elevated levels of *STC2* (*P* = 0.001) and *UBE2C* (*P* = 0.042) had an adverse effect on DSS. For OS, low levels of *BTG2* (*P* = 0.005) and elevated levels of *CBX2* (*P* = 0.005), *STC2* (*P* = 0.001), and *STK32B* (*P* = 0.015) were predictive of outcome. In addition, low S*100A8* mRNA levels (*P* = 0.053) were borderline significant for OS.

For Cohort II, the 16-gene signature was evaluated in RNASeq expression profiling data for 198 OSCC patients. Univariate Cox regression analysis showed that elevated levels of *UBE2C* mRNA levels (*P* = 0.005) were indicative of OS. On the other hand, elevated levels of *NME5* (*P* = 0.050), low *S100A8* levels (*P* = 0.072), and low *SCUBE2* levels (*P* = 0.067) were borderline significant for OS. Furthermore, low *S100A8* levels (*P* < 0.001; log_2_ratio = -2.61) and elevated *UBE2C* levels (*P* = 0.007; log_2_ratio = 0.713) were significantly associated with high histological grade.

### Protein expression levels of the molecular biomarkers in OSCC specimens

Protein expression levels for the candidate biomarkers were evaluated using immunohistochemistry with 45 full-face FFPE specimens representing 43 OSCC patients (Cohort III; Table 1). PIP and DNALI1 were excluded from further analysis due to low expression levels in OSCC samples, whereas LOC389033 was excluded because the gene is not expressed at the protein level. Immunopositivity was shown for all of the examined proteins in peritumoral normal mucous membrane, the salivary glands, and dysplasia, with the exception of AZGP1, SUSD3, UBE2C, and WHSC1L1. AZGP1 and UBE2C were strongly positive in the basal cell layer; SUSD3 was positive in the mucous membrane but negative in the salivary glands, whereas WHSC1L1-positivity was shown in the layers of muscle tissue. In addition, no S100A8 staining was observed in the basal cell layer. In invasive tissue, immunopositivity for the 14 analyzed proteins ranged from 14-86% with CNTNAP2 and WHSC1L1 having the lowest and highest incidence rates, respectively (Table 3). Interestingly, there was only one reported case (6%) of SCUBE2-positivity in lymph node-positive tumors, compared with SCUBE2-positivity in 41% of lymph node-negative tumors. In addition, p16 immunopositivity was observed in 8/43 tumor specimens (19%).

**Table 3:** **Incidence of molecular marker immunopositivity in OSCC** (**Cohort III**)

**Biomarker**	**Total patients (n = 43)**	**Lymph node-positive (n = 16)**	**Lymph node-negative (n = 27)**
AZGP1 cytoplasmic	31 (72%)	12 (75%)	19 (70%)
AZGP1 nuclear	25 (58%)	11 (69%)	14 (52%)
BTG2	16 (37%)	7 (44%)	9 (33%)
CBX2	33 (77%)	15 (94%)	18 (67%)
CNTNAP2	6 (14%)	4 (25%)	2 (7%)
NME5	12 (28%)	6 (38%)	6 (22%)
S100A8	11 (26%)	3 (19%)	8 (30%)
SCUBE2	12 (28%)	1 (6%)	11 (41%)
SERPINA11	17 (40%)	7 (44%)	10 (37%)
STC2	20 (47%)	7 (44%)	13 (48%)
STK32B	15 (35%)	4 (25%)	11 (41%)
SUSD3	25 (58%)	9 (56%)	16 (59%)
UBE2C	8 (19%)	3 (19%)	5 (19%)
WHSC1L1	37 (86%)	14 (88%)	23 (85%)

### Correlation of the molecular biomarkers with clinicopathological features

To investigate whether heterogeneous protein expression of the analyzed antigens is clinically relevant, a correlation analysis was performed with established clinicopathological features (Additional file 2: Table S2). S100A8 was strongly associated with tumor differentiation (*P* = 0.009), e.g. tumors with enhanced S100A8 expression levels were frequently well differentiated (64%) compared with 17% in S100A8-negative tumors. In addition, SCUBE2 was significantly associated with lymph node status (*P* = 0.01), CBX2 with tumor inflammatory infiltration (*P* = 0.03), and STK32B with tumor size (*P* = 0.04). Interestingly, a high proportion of SCUBE2-positive tumors (11/12) were lymph node-negative and all STK32B-positive tumors (15/15) were smaller in size (T1-T2 tumors); minimal peritumoral inflammatory infiltration was found in tumors with reduced CBX2 levels. Additionally, we also found a slight indication that UBE2C and SCUBE2, SERPINA11, and NME5 were associated with tumor differentiation (*P* = 0.06 and *P* = 0.09, respectively), inflammatory infiltration (*P* = 0.09), and p16 expression (*P* = 0.08), respectively.

### Prognostic significance of the molecular biomarkers

**Figure 1:**
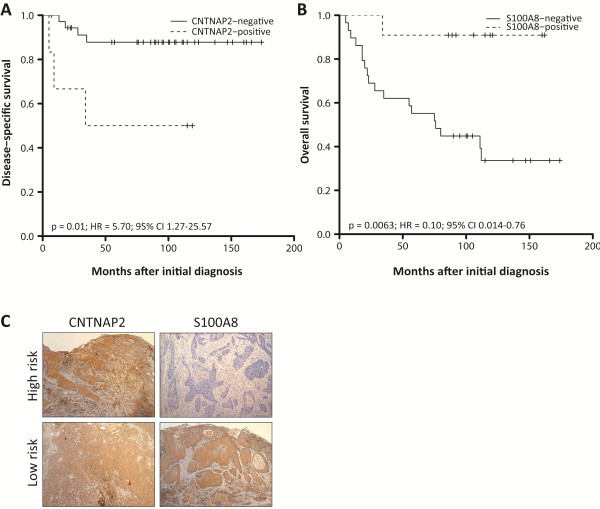
**Prognostic potential of CNTNAP2 and S100A8 protein expression in OSCC. (A**-**B)** Kaplan-Meier estimates of the probability of disease-specific survival and overall survival according to dichotomized protein expression for CNTNAP2 and S100A8, respectively. Patients with CNTNAP2-positive and S100A8-negative tumors had significantly shorter survival times. P-values, hazard ratios (HR), and 95% confidence intervals (95% CI) were calculated using the log-rank test and Cox proportional hazards regression, respectively. The x-axes depict Months after initial diagnosis and the y-axes depict Disease-specific survival or Overall survival. **(C)** Representative immunohistochemical staining showing protein expression patterns in the invasive tissue component.

**Figure 2:**
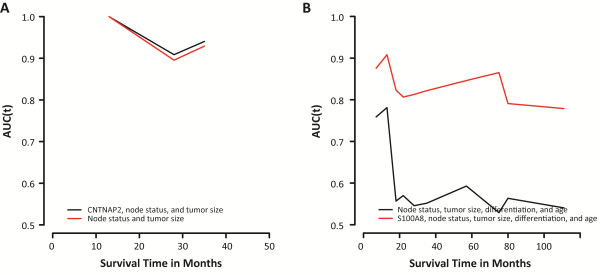
**Predictive performance of prognostic models including CNTNAP2 or S100A8. (A)** The lines represent the time-dependent area under the ROC curve (AUC (t)) for disease-specific survival using established clinical variables (lymph node status and tumor size) assessed individually and in conjunction with CNTNAP2 protein expression. The estimated performance of the model including CNTNAP2 was marginally better than the model containing the established clinical variables alone, increasing the C-index from 0.941 to 0.949. **(B)** The lines represent the time-dependent area under the ROC curve (AUC (t)) for overall survival using established clinical variables (lymph node status, tumor size, differentiation, age) assessed individually and in conjunction with S100A8 protein expression. Combining the established clinical variables with S100A8 protein expression increased the C-index significantly from 0.605 to 0.833. The x-axes depict Survival time in months and the y-axes depict AUC (t).

Next, we examined the prognostic significance of the proposed biomarkers using disease-specific survival and overall survival. OSCC patients with tumors displaying enhanced CNTNAP2 levels had significantly shorter DSS (*P* = 0.010; HR (95% CI) = 5.70 (1.27-25.57)), whereas patients with S100A8-negative tumors had significantly shorter OS (*P* = 0.0063; HR (95% CI) = 0.10 (0.014-0.76); Figure 1). Our data suggest a slight association between SCUBE2 expression and DSS (*P* = 0.090), as well as UBE2C expression and OS (*P* = 0.074). CNTNAP2 expression had no significant effect on DSS after adjusting for tumor size and lymph node status (*P* = 0.10 - HR (95% CI) = 3.56 (0.78-16.17)). Furthermore, outcome prediction was not improved using a predictive model for DSS including CNTNAP2 expression, lymph node status, and tumor size (C-index = 0.949) compared with a model containing lymph node status and tumor size (C-index = 0.941; Figure 2). Following multivariate analysis adjusting for tumor size, lymph node status, differentiation and age, S100A8 was still statistically significant (*P* = 0.013 - HR (95% CI) = 0.11 (0.013-0.92; Table 4)). Combining S100A8 in a predictive model for OS with tumor size, lymph node status, differentiation and age improved outcome prediction significantly from 0.605 to 0.833 (Figure 2).

**Table 4:** **Univariate and multivariate survival analysis for disease**-**specific and overall survival in OSCC** (**Cohort III**)

	**Univariate analysis**					
	**Disease-specific survival**			**Overall survival**		
**Variable**	**HR (95% CI)**	**p**-**value**	**C-index**	**HR (95% CI)**	**p-value**	**C-index**
Age	1.02 (0.96-1.08)	0.58	0.56	**1.05** (**1.01**-**1.09**)	**0.02**	**0.67**
Tumor site	-	0.24	0.67	0.92 (0.31-2.68)	0.87	0.53
Tumor size	**31.59** (**1.57**-**633.64**)	**0**	**0.66**	**15.93** (**1.41**-**179.93**)	**0.02**	**0.58**
Node status	-	**0**	**0.84**	**2.96** (**1.19**-**7.39**)	**0.02**	**0.64**
Differentiation	-	0.11	0.73	0.95 (0.17-5.18)	0.11	0.63
Tumor inflammatory infiltration	3.91 (0.46-33.48)	0.20	0.67	0.91 (0.31-2.72)	0.99	0.51
p16	0.62 (0.074-5.16)	0.65	0.53	0.69 (0.20-2.36)	0.55	0.53
AZGP1 cytoplasmic	0.95 (0.18-4.87)	0.95	0.51	0.88 (0.33-2.32)	0.80	0.52
AZGP1 nuclear	4.57 (0.55-38.01)	0.12	0.65	0.86 (0.35-2.12)	0.75	0.52
BTG2	1.25 (0.28-5.58)	0.77	0.53	0.79 (0.30-2.07)	0.63	0.53
CBX2	-	0.15	0.61	1.12 (0.37-3.37)	0.84	0.51
CNTNAP2	**5.70** (**1.27**-**25.57**)	**0.01**	**0.66**	1.34 (0.39-4.62)	0.65	0.52
NME5	0.37 (0.045-3.11)	0.34	0.58	0.58 (0.19-1.75)	0.33	0.56
S100A8	0.35 (0.043-2.95)	0.32	0.59	**0.10** (**0.014**-**0.76**)	**0.01**	**0.65**
SCUBE2	-	0.090	0.65	1.25 (0.47-3.33)	0.65	0.53
SERPINA11	2.23 (0.50-9.99)	0.28	0.60	0.98 (0.39-2.50)	0.97	0.50
STC2	0.17 (0.023-1.71)	0.10	0.68	0.68 (0.27-1.69)	0.40	0.55
STK32B	0.31 (0.037-2.54)	0.25	0.61	0.47 (0.16-1.41)	0.17	0.58
SUSD3	1.60 (0.31-8.26)	0.57	0.55	0.54 (0.22-1.34)	0.18	0.58
UBE2C	2.56 (0.49-13.28)	0.25	0.57	2.49 (0.88-7.01)	0.074	0.57
WHSC1L1	0.86 (0.10-7.16)	0.89	0.51	1.18 (0.27-5.10)	0.83	0.51
	**Multivariate analysis**					
	**Disease-specific survival**			**Overall survival**		
	**HR (95% CI)**	**p-value**	**C**-**index**	**HR (95% CI)**	**p-value**	**C-index**
CNTNAP2 †	3.56 (0.78-16.17)	0.10	0.95	-	-	-
S100A8 ‡	-	-	-	**0.11** (**0.013**-**0.92**)	**0.01**	**0.83**

*Abbreviations*: *OSCC* oral squamous cell carcinoma, *HR* hazard ratio, *CI* confidence interval, *C*-index concordance index.

Statistically significant data (p < 0.05) indicated with bold text.

† Adjusted for node status and tumor size.

‡ Adjusted for node status, tumor size, differentiation, and age.

## Discussion

Oral squamous cell carcinoma is a heterogeneous disease with diverse clinical, pathological, and biological behavior [10]. Nevertheless, the strongest determinants of prognosis still include tumor stage and the presence of cervical metastases at the time of diagnosis, as well as the time to locoregional recurrences [9,24-26]. Unfortunately, up to 50% of OSCCs are diagnosed at an advanced stage with 5-year survival rates at approximately 60%, e.g. delayed diagnosis [27-30]. Therefore, many patients could benefit greatly from complementary molecular markers, which may help guide treatment decisions and be of value in the development of new therapeutic agents. Extensive efforts are currently being made to identify and validate biomarkers based on the biology of oral cancers that can complement established clinicopathological features and improve clinical management of the disease. Recent work to characterize OSCC using transcriptomic profiling has mainly focused on the identification of biomarkers for disease progression and lymph node metastasis prediction [31-38]. Surprisingly, few gene expression signatures have been developed to improve patient risk assessment [18,34,39,40]. Although transcriptome analyses can give an indication of biological activity within a tissue, mRNA and protein levels may not correlate because gene expression is controlled by a multistage system. We have therefore performed immunohistochemistry using readily available FFPE samples to evaluate the clinical significance of the 16 putative biomarkers in OSCC. Here, we show the prognostic potential of the S100A8 and CNTNAP2 proteins, as well as the relationship between SCUBE2, CBX2, and STK32B protein levels and important clinicopathological features for OSCC, i.e. regional metastasis to the cervical lymph nodes, tumor inflammatory infiltration, and tumor size, respectively.

In the present work, we evaluated the applicability of breast cancer prognostic biomarkers for OSCC, given the biological similiarities between the two cancer types [10]. In breast carcinoma, we showed the recurrent upregulation of the *CBX2*, *CNTNAP2*, *S100A8*, *UBE2C*, and *WHSC1L1* genes as well as downregulation of the *AZGP1*, *BTG2*, *DNALI1*, *LOC389033*, *NME5*, *PIP*, *SCUBE2*, *SERPINA11*, *STC2*, *STK32B*, and *SUSD3* genes in more aggressive tumors [14,15,23]. The prognostic potential of individual biomarkers in the 16-gene signature were evaluated in three OSCC patient cohorts containing clinical information, two of which (Cohorts I-II) were compiled from publicly available Affymetrix and Illumina RNASeq datasets. The Affymetrix one-channel system and Illumina RNAseq platform have recently been shown to correlate well, in particular for high abundance genes [41]. Although the majority of the markers in the signature were in agreement for both breast carcinoma and OSCC, five proteins (S100A8, STC2, STK32B, SUSD3, and WHSC1L1) were inversely regulated in aggressive OSCC samples. In addition, several of the biomarkers which showed promise at the mRNA level (*CBX2*, *S100A8*, *SCUBE2*, and *STK32B*) were also either predictive of clinical outcome or associated with clinicopathological features at the protein level. The differences in the prognostic potential of specific biomarkers at the mRNA or protein levels may possibly be the result of small sample sizes, discordant mRNA-protein expression patterns or differences in sample preparation for microarray analyses and immunohistochemistry, i.e. microarray is frequently performed using a tumor mass, which consists of both malignant and nonmalignant cells, resulting in an over- or underestimation of expression levels, whereas cell type-specific protein expression patterns can be easily interpreted using immunohistochemistry. In breast carcinoma, elevated levels of the S100A8 protein were associated with moderate/strong tumor inflammatory infiltration and significantly shorter DSS rates. In contrast, lower S100A8 protein levels were associated with significantly shorter OS rates in OSCC, but there was no association with tumor infiltration. These findings suggest differences in cell type-specific gene expression and thereby activation and/or inhibition of diverse cellular mechanisms. Furthermore, CBX2, CNTNAP2, S100A8, SCUBE2, and STK32B protein levels were significantly associated with established clinicopathological features for OSCC and/or patient clinical outcome. We propose here two potential prognostic biomarkers for OSCC (the CNTNAP2 protein for DSS and the S100A8 protein for OS). There may be several reasons why these candidate biomarkers were associated with different clinical endpoints: differences in gene function and whether the biomarkers correlated with strong determinants of OSCC-related prognosis, e.g. tumor stage, lymph node metastasis or time to locoregional recurrences.

Chen *et al*. identified upregulation of *CNTNAP2* mRNA levels as a distinctive characteristic of OSCC in comparison with normal mucosa [32]. In a genome-wide association study for oral cancers, *CNTNAP2* was found to be associated with cell migration [42]. The *CNTNAP2* gene also functions as a cell adhesion molecule and has been found to be either methylated or deleted in several different cancer types, e.g. glioma, myleoid leukemia, and pancreatic adenocarcinoma [43-45]. In the present study, elevated CNTNAP2 protein levels were prevalent in tumors from pN1 patients, but this association was not statistically significant likely due to the low number of events. These findings and the known function of the CNTNAP2 protein in tumorigenesis suggest that it may be a determinant of metastatic spread to the cervical lymph nodes and thereby a more aggressive phenotype with an adverse effect on DSS. Although elevated CNTNAP2 levels were predictive of a more unfavorable prognosis in univariate Cox regression models, disease-specific survival rates were not significantly improved by including the protein in a model containing lymph node status and tumor size. These results show the profound effect node metastases have on clinical outcome. In future studies, it will be necessary to alter the study design to test the prognostic potential of CNTNAP2 further, e.g. using a larger cohort containing node-negative patients. Furthermore, the *S100A8* gene, also known as MRP-8 or calgranulin A, has been studied extensively in head and neck cancer. Inclusion of S100A8 protein expression patterns in multivariate models together with tumor size, node status, and age significantly improved OS prediction. In addition, we found an association between low S100A8 levels (mRNA levels in Cohort II and protein levels in Cohort III) and tumor differentiation. *S100A8* belongs to the S100 gene family, several of which form a gene cluster on chromosome 1q21 and are commonly induced by chronic and acute inflammation [46-48]. In the present study, positive S100A8 immunostaining was also observed in a peritumoral inflammatory cell population, albeit similar in both S100A8-positive and negative tumors. Overexpression of the S100A8 protein has been observed in breast, colorectal, gastric, lung, pancreatic, and prostate cancer, wheras underexpression has been shown in various squamous cell carcinomas of the head and neck [49]. Recently, the S100A8/A9 heterocomplex was identified as a regulator of cell cycle progression and cell proliferation in cancer cell lines originating from the head and neck region [50].

OSCC outcome prediction may also be improved by identifying biomarkers that can predict which tumors will inevitabily spread to the cervical nodes. When we initiated this study, we believed that the *CNTNAP2* gene would be a strong candidate for lymph node status because of its association with metastatic spread to the axillary lymph nodes in breast carcinoma [23]. However in the present study, loss of SCUBE2 protein expression in the tumor epithelial component was the only marker significantly associated with lymph node status and borderline significant for DSS. Interestingly, all 12 patients with SCUBE2-positive tumors were long-term disease-specific survivors and only 1/12 tumors had metastasized to the cervical lymph nodes.These findings are consistent with reports in breast carcinoma where elevated levels of the SCUBE2 protein not only inhibited proliferation of breast cancer cell lines, but was indicative of a more favorable prognosis [23,51]. SCUBE2 is a multidomain, secreted glycoprotein that was first implicated in functioning upstream of hedgehog signaling in zebrafish, but has since been shown to also play a pivotal role in human tumorigenesis [52,53]. Furthermore, SCUBE2 serves two different functions by using either the CUB domain at the C-terminal end or the EGF-like repeats at the N-terminus, i.e. control of cell proliferation by antagonizing bone morphogenetic protein (BMP) activity and regulation of cell-cell contacts, and possibly tumor metastasis, by forming complexes with E-cadherin in adherens junctions, respectively [54].

The role CBX2 and STK32B play in OSCC tumorigenesis is largely unknown. In a correlation analysis between protein expression patterns and established clinicopathological features, a clear link was found between CBX2-positivity and tumor inflammatory infiltration as well as STK32B-positivity and small tumor size (T1-T2). CBX2 plays a role in epigenetic regulation and hematopoietic stem cell differentiation, whereas the *STK32B* gene is one of several serine/threonine kinases (*STK32B*, *STK36*, and *STK39*) with similar gene expression patterns in basal-like breast cancers [16,55,56]. Consequently, *STK36* is a component of the hedgehog signaling pathway. The function of the *STK32B* gene is not known, but single nucleotide polymorphisms in the *STK32B* gene have been associated with oral clefts [57].

Hedgehog signaling has also been implicated in OSCC and basal cell carcinoma, a skin cancer frequently found in the head and neck region [58,59]. Leovic and colleagues illustrated the clinical relevance and aberrant expression of key hedgehog signaling components in SCCs of the oral cavity and oropharynx, i.e. *PTCH1*, *GLI1*, *SMO*, and *SHH*[58]. In Cohort II, several genes which play a role in hedgehog signaling were also found to be associated with high histological grade (the *KIF7*, *TCTN1*, and *TCTN2* genes) and metastatic spread to the cervical lymph nodes (the *FGF4* and *PTCHD1* genes). These findings further emphasize the importance of hedgehog signaling in OSCC.

## Conclusions

In summary, our results illustrate that cancer is a complex, heterogeneous disease. However, certain clinically relevant biomarkers may be useful therapeutic targets for several different cancer types. Despite the limited number of patients included in the study, the integrative analysis identified at least five of the putative biomarkers originally developed in breast carcinoma (CBX2, CNTNAP2, S100A8, SCUBE2, and STK32B) that also improve risk assessment for OSCC patients and may play a crucial role in cancer-related processes, e.g. hedgehog signaling, regulation of cell cycle progression, cell proliferation, metastasis. These targets should be studied in a larger cohort to further evaluate their clinical significance in OSCC.

## Abbreviations

OSCC: squamous cell carcinoma of the oral cavity; DSS: Disease-specific; OS: Overall survival; pN1: cervical lymph node metastases; pN0: neck negative; pNmic: micrometastasis; H & E: Hematoxylin and eosin; FFPE: Formalin-fixed, paraffin-embedded tumor tissues; C-index: Concordance index; AUC (t): time-dependent area under the ROC curve.

## Competing interests

The authors declare that they have no competing interests.

## Authors’ contributions

TZP, LA, AK, CYC, PK, KH contributed to the study design. TZP, SH, MS, AK performed the immunohistochemical analyses. TZP performed the data and statistical analyses, and drafted the manuscript. LA collected clinical data, performed the evaluation of lymph nodes, data analyses, and drafted the manuscript. AK performed the pathologic review of the FFPE samples. SN performed the statistical analyses. AK, SH, SN, MS, CYC, PK, KH critically reviewed the manuscript. All authors have read and approved the final manuscript.

## Pre-publication history

The pre-publication history for this paper can be accessed here:

http://www.biomedcentral.com/1471-2407/14/324/prepub

## Supplementary Material

Additional file 1: Table S1 Optimal antibody conditions and H-score cutoffs. Click here for file

Additional file 2: Table S2 Relationship between protein expression and clinicopathological features in OSCC (Cohort III). Click here for file
